# Severe leukoencephalopathy with fulminant cerebral edema reflecting immune reconstitution inflammatory syndrome during HIV infection: a case report

**DOI:** 10.1186/1752-1947-4-214

**Published:** 2010-07-17

**Authors:** Christian Oelschlaeger, Rainer Dziewas, Doris Reichelt, Jens Minnerup, Thomas Niederstadt, Erich B Ringelstein, Ingo W Husstedt

**Affiliations:** 1 University Hospital Muenster, Department of Neurology, A.-Schweitzer-Str. 33, D-48129 Muenster, Germany; 2 University Hospital Muenster, Department of Internal Medicine, A.-Schweitzer-Str. 33, D-48129 Muenster, Germany; 3 University Hospital Muenster, Institute for Clinical Radiology, A.-Schweitzer-Str. 33, D-48129 Muenster, Germany

## Abstract

**Introduction:**

Immune reconstitution inflammatory syndrome is a well-known complication in HIV-infected patients after initiation of highly active antiretroviral therapy resulting in rapid CD4^+ ^cell count recovery and suppression of viral load. Generally, immune reconstitution inflammatory syndrome is based on opportunistic infections, but rare cases of immune reconstitution inflammatory syndrome inducing demyelinization of the nervous system have also been observed.

**Case presentation:**

A 37-year-old African woman with HIV infection diagnosed at 13 years of age was admitted to the emergency department after experiencing backache, severe headache, acute aphasia and psychomotor slowing for one week. Nine weeks earlier, highly active antiretroviral therapy in this patient had been changed because of loss of efficacy, and a rapid increase in CD4^+ ^cell count and decrease of HIV viral load were observed. Magnetic resonance imaging of the brain showed extensive white matter lesions, and analysis of cerebrospinal fluid revealed an immunoreactive syndrome. Intensive investigations detected no opportunistic infections. A salvage therapy, including osmotherapy, corticosteroids and treatment of epileptic seizures, was performed, but the patient died from brainstem herniation 48 hours after admission. Neuropathologic examination of the brain revealed diffuse swelling, leptomeningeal infiltration by CD8 cells and enhancement of perivascular spaces by CD8+ cells.

**Conclusion:**

Immune reconstitution inflammatory syndrome in this form seems to represent a severe autoimmunologic disease of the brain with specific histopathologic findings. This form of immune reconstitution inflammatory syndrome did not respond to therapy, and extremely rapid deterioration led to death within two days. Immune reconstitution inflammatory syndrome may also occur as severe leukoencephalopathy with fulminant cerebral edema during HIV infection with rapid immune reconstitution.

## Introduction

The benefit of highly active antiretroviral therapy (HAART) in HIV-infected patients is the restoration of the immune system. According to the literature, up to 37% of these patients may develop an immune reconstitution inflammatory syndrome (IRIS) mainly when HAART is started in antiretroviral-naïve patients, who develop a rapid recovery of immune function [1-4]. IRIS may develop based on opportunistic infections (e.g., with *Mycobacterium tuberculosis*, *Cryptococcus neoformans *or JC-virus inducing progressive multifocal leukoencephalopathy). IRIS of the peripheral or central nervous system as Guillain-Barré syndrome or leukoencephalopathy has also been observed [1-6].

## Case presentation

A 37-year-old African woman with HIV infection diagnosed at age 13 years was admitted to the emergency department after experiencing backache, severe headache, acute aphasia and psychomotor slowing for one week. Nine weeks before admission to the hospital, HAART had been changed in this patient from 500 mg/d of zidovudine, 300 mg/d of lamivudine and 200 mg/d of emtricitabine to the fixed preparation of lopinavir/ritonavir (800 mg/200 mg/d) and saquinavir (2000 mg/d). HAART was necessary for 10 years in this patient and was started at a level of 255 CD4^+ ^cells/mm^3^. Before admission to the hospital, the patient's CD4^+ ^cell count had rapidly increased from 20 to 610 cells/mm^3^, and her HIV-1 viral load decreased from 63,200 to 120 c/mL. Twelve hours after admission, the patient developed severe psycho-organic syndrome and repeated generalized epileptic seizures. Therefore, anticonvulsive therapy with levetiracetam was initialized. Computed tomography of the skull showed generalized cerebral edema without focal lesions. Intensive osmotherapy using mannitol and glycerol to minimize cerebral edema was performed. Blood tests revealed normal C-reactive protein and a normal white blood cell (WBC) count; radiographs of the chest were normal. Analysis of cerebrospinal fluid (CSF) showed pleocytosis (36 WBC/mm^3^), an elevated protein level of 1120 mg/dL, a normal glucose level of 61 mg/dL and no oligoclonal bands. CSF microscopy yielded no bacteria or fungi. Virus polymerase chain reaction (PCR) results for JC-virus (JCV), herpes simplex virus (HSV), cytomegalovirus (CMV), Epstein-Barr virus (EBV), varicella zoster virus (VZV) and hepatitis B virus using whole blood were negative. PCR of the CSF did not detect JCV, HSV, CMV, EBV, VZV or Tuberculosis.

**Figure 1:**
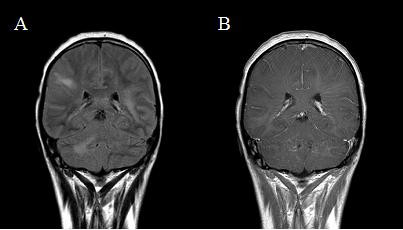
**Magnetic resonance imaging (MRI) scans**. This MRI scan from the patient's initial presentation shows a perivascular infiltration and intraparenchymal, large-scale lesions in both hemispheres on the T2-weighted images (**A**). Application of gadolinium revealed no enhancement of the lesions in the T1-weighted images (**B**).

Twenty-four hours later, the patient fell comatose and needed mechanical ventilation. Magnetic resonance imaging (MRI) of the brain showed parietal and occipital hyperintense white matter lesions on T2-weighted images on both sides. Using contrast medium, these lesions did not reveal any enhancement in the T1-weighted images. However, there was a strong enhancement in perivascular spaces (Figure 1). As salvage therapy, 1 g/d of methylprednisolone and the antimicrobial agents ampicillin, acyclovir, ceftriaxone and fluconazole were applied. Without any improvement, the patient died from brainstem herniation two days after admission.

**Figure 2:**
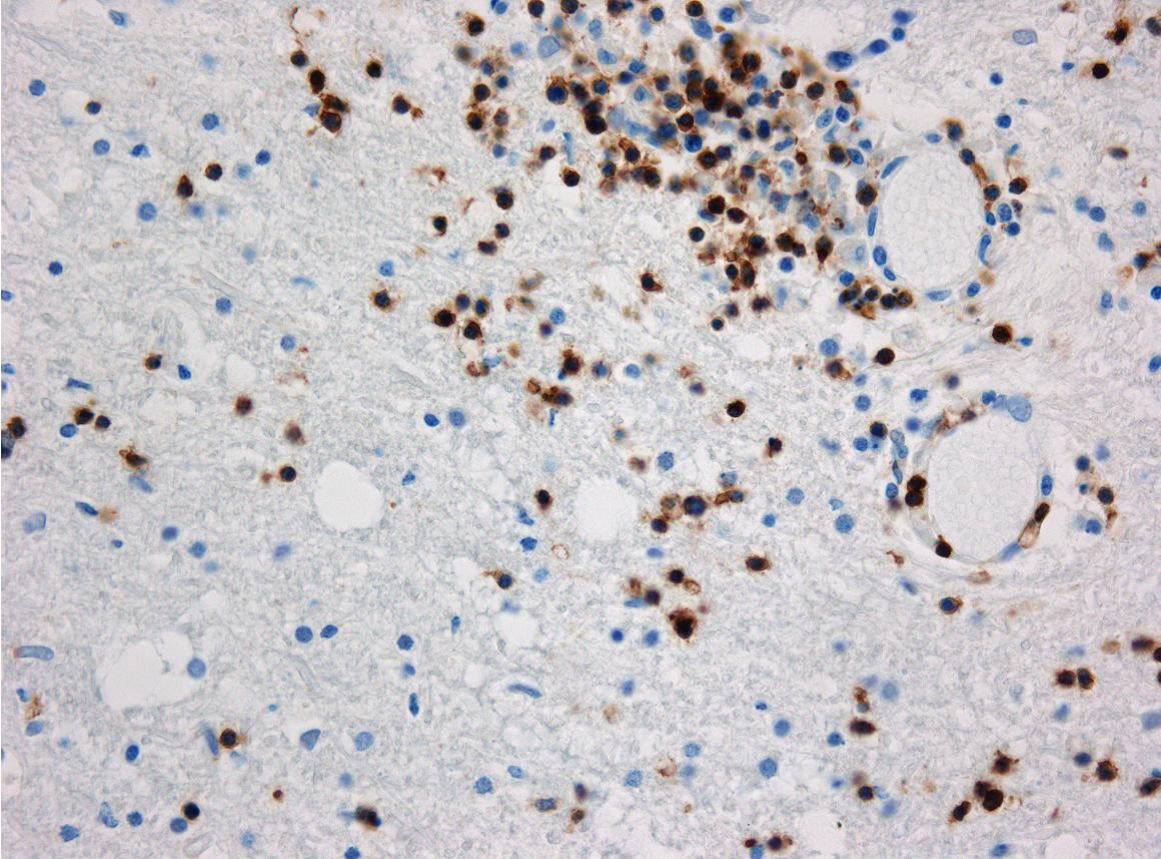
**Histopathology**. On histopathologic examination, lymphocytic infiltrates are encountered around cerebral blood vessels and in the white matter. Immunohistochemistry for CD3 confirms the T-cellular character of the inflammatory infiltrates (original magnification ×400).

With the patient's relatives' consent, autopsy of the brain was performed. The post-mortem study of the brain showed a diffuse swelling of the whole brain and leptomeningeal infiltration with lymphocytes. These changes were also documented by MRI and represented the explanation for the epileptic seizures. Severe inflammatory infiltration in perivascular spaces by T-lymphocytes of the CD8^+ ^subtype was found (Figure 2). Reactive astrocytes were found in the white matter, but no macrophages were seen. Results of Gram, periodic acid-Schiff and Grocott staining and immunocytochemistry for CMV and toxoplasma were negative. Congestion bleeding in the brainstem was the result of herniation, leading to death.

## Discussion

A detailed case report of severe lethal leukoencephalopathy presumably an autoimmunologic manifestation of IRIS proven by typical neuropathologic results and corresponding MRI results is presented. IRIS usually starts after the initiation of HAART. The main risk factor for IRIS represents rapid immune restoration from a high viral load and low CD4^+ ^cell count at the beginning of HAART [1-4,7]. Recovery of lost CD4^+ ^cells initiates a complex immune cascade, inducing IRIS. CD4^+ ^cells trigger cytotoxic reaction of CD8^+ ^cells against antigens and may cause vasculitis, cerebritis or both [4,8]. The increase in circulating newly developed CD4^+ ^cells triggers abnormal immune responses by CD8^+ ^dysfunction in different organs such as the liver, eyes, lungs and brain [1-4,8]. IRIS is well known during opportunistic infections (e.g., toxoplasmosis, progressive multifocal leukoencephalopathy). IRIS manifesting as Guillain-Barré syndrome (GBS) or severe leucencephalopathy occurs only rarely [1,4-6,9]. Neuropathologic examination revealed perivascular infiltration of T-lymphocytes predominantly of the CD8^+ ^subtype in the brain and enhancement of perivascular spaces. Both pathologic findings have been described as irregular immune responses during IRIS [9]. Diffuse infiltrative lymphocytosis syndrome as an uncommon manifestation characterized by persistent circulating CD8^+ ^lymphocytosis in salivary glands may represent a less severe manifestation of this form of IRIS, and infiltration of a brain by CD8^+ ^cells is proven in this case by neuropathologic examination [5,8]. Because no opportunistic infection could be detected, the autoimmunologic cause of IRIS is most likely and is demonstrated by neuropathologic results. Although wide pharmacologic therapy with corticosteroids and antimicrobial agents as salvage therapy was performed, the patient died two days after admission, showing the ineffectiveness of therapies that are generally effective in other forms of IRIS. The benefit of corticosteroid therapy has been described in patients with IRIS after opportunistic infection and is also an established therapy in patients with GBS [1,3,10]. In contrast to these results, corticosteroids were not successful in this fulminant form of IRIS presenting as severe, rapid, progressive leukoencephalopathy.

Up until now, diagnostic and therapeutic standards for IRIS have not been precise, and a clear classification and guidelines for therapeutic strategies in these new manifestations of IRIS are absolutely necessary. In every case, IRIS represents a severe complication of excellent immune restoration and should be considered in rapidly deteriorating patients. It represents a big diagnostic problem because predictors indicating early-stage IRIS are still unknown.

## Conclusion

If distinct neurologic symptoms appear in HIV-1-infected patients after initiation of HAART and initial clinical improvement, central nervous system IRIS should be considered. Application of corticosteroids represents a reasonable therapy for patients with mild cerebral edema but may fail in those with severe, rapid, progressive cases.

## Consent

Written informed consent was obtained from the patient's next-of-kin for publication of this case report and any accompanying images. A copy of the written consent is available for review by the Editor-in-Chief of this journal.

## Competing interests

The authors declare that they have no competing interests.

## Authors' contributions

CO was responsible for the medical care of the patient and was the major contributor in writing the manuscript, RD was the head of the intensive care unit, JM was the responsible for the medical care in the intensive care unit, TN analyzed the radiologic examination, EBR is the department chair and IWH was the head of the division of neuro-AIDS and was a contributor in writing the manuscript.

All authors read and approved the final manuscript.
